# Modelling Seasonal Brucellosis Epidemics in Bayingolin Mongol Autonomous Prefecture of Xinjiang, China, 2010–2014

**DOI:** 10.1155/2016/5103718

**Published:** 2016-10-30

**Authors:** Pengwei Lou, Lei Wang, Xueliang Zhang, Jiabo Xu, Kai Wang

**Affiliations:** ^1^College of Public Health, Xinjiang Medical University, Urumqi 830011, China; ^2^College of Medical Engineering and Technology, Xinjiang Medical University, Urumqi 830011, China; ^3^Department of Basic Teaching, Xinjiang College of Engineering, Urumqi 830000, China

## Abstract

Brucellosis is one of the severe public health problems; the cumulative number of new human brucellosis cases reached 211515 from 2010 to 2014 in China. Bayingolin Mongol Autonomous Prefecture is situated in the southeast of Xinjiang, where brucellosis infection occurs every year. Based on the reported data of newly acute human brucellosis cases for each season in Bayingolin Mongol Autonomous Prefecture, we proposed a susceptible, exposed, infected, and vaccinated (SEIV) model with periodic transmission rates to investigate the seasonal brucellosis transmission dynamics among sheep/cattle and from sheep/cattle to humans. Compared with the criteria of MAPE and RMSPE, the model simulations agree to the data on newly acute human brucellosis. We predict that the number of newly acute human brucellosis is increasing and will peak 15325 [95% CI: 11920–18242] around the summer of 2023. We also estimate the basic reproduction number *R*_0_ = 2.5524 [95% CI: 2.5129–2.6225] and perform some sensitivity analysis of the newly acute human brucellosis cases and the basic reproduction number *R*_0_ in terms of model parameters. Our study demonstrates that reducing the birth number of sheep/cattle, raising the slaughter rate of infected sheep/cattle, increasing the vaccination rate of susceptible sheep/cattle, and decreasing the loss rate of vaccination are effective strategies to control brucellosis epidemic.

## 1. Introduction

Brucellosis is a contagion-allergy zoonosis, it is caused by Gram-negative bacteria of the genus* Brucella* which includes* Brucella abortus*,* Brucella melitensis*,* Brucella suis*,* Brucella neotomae*,* Brucella ovis*, and* Brucella canis* [1, 2]. British military doctor Bruce was the first to confirm the pathogen of the disease in 1886; hence the disease was named “brucellosis” in order to honor him [3, 4]. Brucellosis primarily affects cattle and sheep, which also infects dogs, elks, swine, horses, and humans. It firstly spreads among animals and then transmits to humans. In animals, brucellosis mainly damages the reproductive system and results in abortion and sterility. In humans, it can lead to the symptoms of fever, wandering arthritis, liver, spleen, and lymph node enlargement, testicular pain and swelling, neuralgia, and so forth. After the mid-20th century, the controlled brucellosis resurrects in most parts of the world, especially in the Mediterranean areas, the Middle East, South and Central America, Asia, and so forth [5]. It has spread so widely that there are 123 countries where brucellosis has occurred around the world. The incidence of brucellosis rose sharply in many countries. At present, approximately 0.5 million new brucellosis cases annually are estimated by the World Health Organization (WHO) to occur globally [6].

In China, the five major pastoral areas of brucellosis infection are located in Inner Mongolia, Xinjiang, Tibet, Qinghai, and Ningxia, among which, Inner Mongolia is the most serious region since 2004 [7]. According to* China Statistical Yearbook*, the numbers of new human brucellosis cases in 2012, 2013, and 2014 were 39151 (2.93/100000), 43468 (3.21/100000), and 57222 (4.22/100000), respectively. And according to* Xinjiang Statistical Yearbook*, the numbers of new human brucellosis cases in 2012, 2013, and 2014 were 2335 (9.80/100000), 4095 (17.51/100000), and 7358 (33.02/100000), respectively. We can infer from the reported data of new human brucellosis cases in China and Xinjiang that brucellosis is becoming increasingly serious.

It is well known that whooping cough, measles, influenza, polio, chickenpox, mumps, and so forth exhibit seasonal fluctuations [8–10]. In China, it is generally acknowledged that from January to March is spring, from April to June is summer, July to September is autumn, and October to December is winter [11]. As we all know that brucellosis has seasonal variations, hence, the numbers of new human brucellosis cases are significantly increasing annually in summer and autumn, while the numbers in spring and winter are relatively decreasing [12].

**Figure 1:**
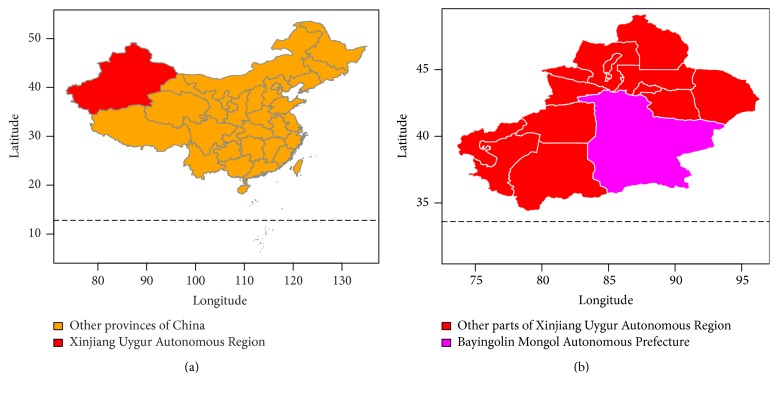
The specific geography location of Bayingolin Mongol Autonomous Prefecture. (a) The map of China. (b) The map of Xinjiang Uygur Autonomous Region.

**Figure 2:**
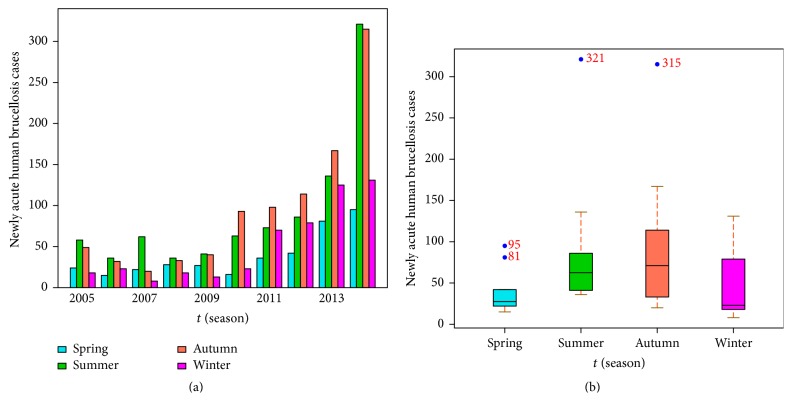
The reported data of newly acute human brucellosis cases in each season from 2005 to 2014. (a) The reported data are described with a bar diagram. (b) The reported data are displayed with a boxplot.

**Figure 3:**
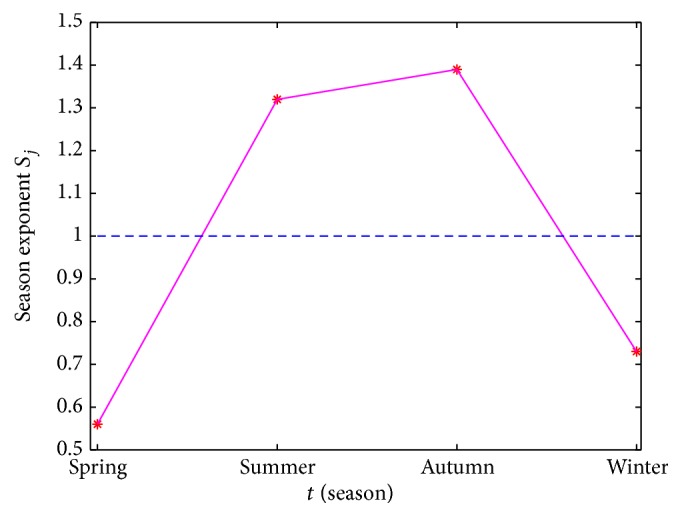
The season exponent of average newly acute human brucellosis cases from 2005 to 2014. The asterisks represent the season exponent values.

This paper focuses on the epidemic dynamics of brucellosis in Bayingolin Mongol Autonomous Prefecture. Bayingolin Mongol Autonomous Prefecture belongs to the Xinjiang Uygur Autonomous Region of China, which is one of the major livestock breeding areas, and the specific geography locations are presented in Figures 1(a) and 1(b), where brucellosis infection occurs every year, and even an outbreak during the summer and autumn of 2014 appeared. We utilize the data of newly acute human brucellosis cases in each season from 2005 to 2014 reported by the Center for Disease Control and Prevention of Bayingolin Mongol Autonomous Prefecture in Xinjiang, and then we plot a bar diagram and a boxplot which are presented in Figures 2(a) and 2(b), respectively. In statistics, season exponent *S*_*j*_  (*j* = 1,2, 3,4) reflects a stable relationship between the average number of newly acute human brucellosis cases ${\bar{x}}_{j}$ and the average number of total newly acute human brucellosis $\bar{x}$ [13, 14]. If *S*_*j*_ > 1, it indicates that ${\bar{x}}_{j}$ is higher than $\bar{x}$; if *S*_*j*_ < 1, it demonstrates that ${\bar{x}}_{j}$ is lower than $\bar{x}$; if *S*_*j*_ ≈ 1, it manifests that the newly acute human brucellosis does not present an obvious seasonal effect. Eventually, from Table 1 and Figure 3 we can conclude that the numbers of newly acute human brucellosis cases in spring and winter are less than those in summer and autumn. Thus, it can be confirmed that the newly acute human brucellosis cases in Bayingolin Mongol Autonomous Prefecture show a pronounced seasonal fluctuation.

The factors which influence human brucellosis seasonal trend can be shown as follows.

1. In the early summer and late autumn, the suitable temperature and weather provide a favorable environment for sheep mating and breeding. As a matter of fact, sheep are capable of breeding once or twice a year, mainly in early April and late September, and the average gestation period is around 150 days. The length of estrus and mating period for cattle is approximately from July to September, and the average gestation period is around 285 days; thus breeding season is delayed to the second year starting in May until July. In conclusion, these increase the contact rates between humans and the secretions, abortuses, viscera, skin, fur, and so forth all from the infected sheep/cattle, eating undercooked meat, drinking raw milk, or being exposed to the contaminated environment under the circumstance with absence of protective measures. Consequently, the risk of human infection with brucellosis from infected sheep/cattle in the summer and autumn is enhanced [3, 15].
2. Temperature has a significant influence on the activity of* Brucella*. Studies have confirmed that under the temperature of 37°C demonstrated the peak breeding activity [16]. Accordingly, the higher temperature in the summer and autumn contributes to the higher activity of* Brucella* compared with spring and winter.

Different mathematical models have been developed to investigate the transmission dynamics of brucellosis among cattle, sheep, elk, and human [17–22]. For instance, Hou et al. [23] proposed an SEIVB dynamic model for the sheep-human transmission of brucellosis considering the impact of* Brucella* in the environment and vaccination for the susceptible sheep on brucellosis transmission and used the model to simulate the brucellosis data in Inner Mongolia of China. Li et al. [24] developed a deterministic model to investigate the transmission dynamics of brucellosis in Hinggan League of Inner Mongolia, China. In addition, they compared the effect of existing mixed cross infection between basic ewes and other sheep on the newly infected human brucellosis cases. Dobson and Meagher [25] employed a simple SIR epidemiological model to describe the population and disease dynamics of brucellosis among bison and elk in the Greater Yellowstone Area. Nevertheless, none of these studies analyzed the brucellosis with seasonal fluctuation.

We refer to the other published articles which are related to the periodic diseases transmission models [26–30]. Particularly, Zhang et al. [29] proposed a SEIRS model with periodic transmission rates to investigate the seasonal rabies epidemics in China and demonstrated that it was more reasonable to regard basic reproduction number *R*_0_ rather than the average basic reproduction number ${\tilde{R}}_{0}$ or the basic reproduction number ${\hat{R}}_{0}$ of the corresponding autonomous system as a threshold for the disease. Ma et al. [30] established an SEII_*e*_QR epidemic model with periodic transmission rate to study the spread of seasonal HFMD in Shandong Province and analyzed the dynamical behaviors of the model.

The purpose of this paper is to develop a periodic brucellosis transmission model among sheep/cattle and from sheep/cattle to humans in the Bayingolin Mongol Autonomous Prefecture of Xinjiang, China. We firstly use the model to simulate the data of newly acute human brucellosis cases reported by the Center for Disease Control and Prevention of Bayingolin Mongol Autonomous Prefecture from the spring of 2010 to the winter of 2014 and then determine the basic reproduction number and analyze the dynamic behaviors of the model. Some sensitivity analyses of the newly acute human brucellosis cases and the basic reproduction number *R*_0_ in terms of some key parameters are carried out. Finally, we explore some effective strategies for the brucellosis in Bayingolin Mongol Autonomous Prefecture.

The article is organized as follows. In Section 2, we introduce the brucellosis transmission model, accounting for the parameters and the functions of periodic transmission rates. In Section 3, the globally asymptotic stability of the disease-free equilibrium is studied. The numerical simulations, prediction of the epidemic trends for the next decades, estimation of the basic reproduction number, and sensitivity analysis of the basic reproduction number and the newly acute human brucellosis cases are presented in Section 4. In Section 5, we put forward various control measures and give a brief discussion.

## 2. Brucellosis Model Formulation and Analysis

**Figure 4:**
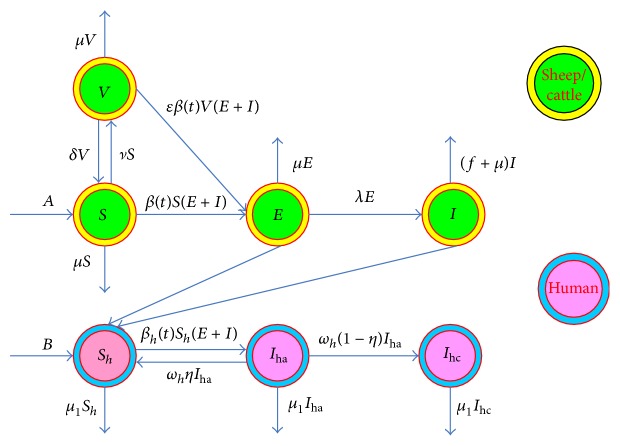
The flowchart of brucellosis transmission among sheep/cattle and from sheep/cattle to humans.

In order to establish the brucellosis transmission model between sheep/cattle and humans, we divide the sheep/cattle population into four subclasses: the susceptible, the exposed, the infected, and the vaccinated, denoted by *S*(*t*), *E*(*t*), *I*(*t*), and *V*(*t*), respectively. The human population is divided into three subclasses: the susceptible, the acute infected, and the chronic infected, denoted by *S*_*h*_(*t*), *I*_ha_(*t*), and *I*_hc_(*t*), respectively. The mean incubation period of human brucellosis is about two weeks, infected patients mainly have fever during this period, they only take some medicine for the treatment of fever which was mistaken as the common cold, and after showing clinical symptoms then they would go to hospital for checking. In the meantime, human brucellosis has reached an acute infection status; thus we assume that the susceptible people infected with brucellosis will directly enter into the acute infection compartment. The flowchart of brucellosis transmission is illustrated in Figure 4.

The model is described as the following ordinary differential equations:

(1) dSdt=A−βtSE+I−μ+νS+δV,dEdt=βtS+ϵVE+I−λ+μE,dIdt=λE−μ+fI,dVdt=νS−μ+δV−ϵβtVE+I,dShdt=B−βhtShE+I+ωhηIha−μ1Sh,dIhadt=βhtShE+I−ωhIha−μ1Iha,dIhcdt=ωh1−ηIha−μ1Ihc.

All parameters are assumed positive. We need to interpret the parameters that appear in our model. The birth numbers of sheep/cattle and humans per unit time are constants and denoted by *A* and *B*, respectively. Regarding the parameters for sheep/cattle, *ν* and *ϵ* are the products of the vaccination rate and the invalid vaccination rate; *δ* represents the loss rate of vaccination; the transfer rate from the exposed to the infected can be identified as *λ*; the slaughter rate is attributed to being infected with brucellosis and the natural mortality rate which are indicated as *f* and *μ*, respectively. For the human population, *ω*_*h*_ *η* is the cure rate from the acute infection to the susceptible; we assume that all patients who are not healed in acute infection will progress into chronic infection; thus the transfer rate from acute infection to chronic infection is *ω*_*h*_(1 − *η*); *μ*_1_ is the natural death rate; since the mortality rate of human brucellosis is quite low, it can be negligible.

The functions *β*(*t*) and *β*_*h*_(*t*) can be expressed as *β*(*t*) = *α*[1 + *b*sin⁡((*π*/2)*t* + *c*)] and *β*_*h*_(*t*) = *α*_*h*_[1 + *b*_*h*_sin⁡((*π*/2)*t* + *c*_*h*_)] proposed by Schenzle [32] to describe the transmission rates among sheep/cattle and from sheep/cattle to humans, respectively, where *α* and *α*_*h*_ are the baseline contact rates, *b* and *b*_*h*_ are the magnitudes of forcing, and *c* and *c*_*h*_ are the phase. The above six parameters are constants, which can be estimated by the least-square fitting and bootstrap method in Section 4.

The first four equations are independent of the last three equations in model (1), and thus we can only consider the first four equations:

(2) dSdt=A−βtSE+I−μ+νS+δV,dEdt=βtS+ϵVE+I−λ+μE,dIdt=λE−μ+fI,dVdt=νS−μ+δV−ϵβtVE+I.

It is easy to see that model (2) has a unique positive disease-free equilibrium *P*_0_ = (*S* ^*∗*^, 0,0, *V* ^*∗*^), where

(3) S∗=Aμ+δμμ+ν+δ,V∗=Aνμμ+ν+δ.

Consider the following auxiliary equations:

(4) dxdt=A−μ+νx+δy,dydt=νx−μ+δy.

**Lemma 1 .**

Model (4) has a unique positive globally asymptotically stable equilibrium (*A*(*μ* + *δ*)/*μ*(*μ* + *ν* + *δ*), *Aν*/*μ*(*μ* + *ν* + *δ*)).

**Proof**

In fact, the Jacobian matrix of model (4) at equilibrium (*A*(*μ* + *δ*)/*μ*(*μ* + *ν* + *δ*), *Aν*/*μ*(*μ* + *ν* + *δ*)) is

(5) $$\begin{array}{c} J=\left(\begin{array}{cc} -\left(\mu +\nu \right) & \delta \\ \nu & -\left(\mu +\delta \right) \end{array}\right) \end{array}$$

and then the corresponding characteristic equation is

(6) $$\begin{array}{c} \Phi \left(\;\lambda \;\right)=\lambda^{2}+\left(\;2\mu +\delta +\nu \;\right)\lambda +\left(\;\mu +\nu +\delta \;\right)\mu . \end{array}$$

By simple calculation, it is easy to obtain that the two roots of Φ(*λ*) are *λ*_1_ = −*μ* and *λ*_2_ = −(*μ* + *δ* + *ν*). Hence, we obtain that the unique positive equilibrium (*A*(*μ* + *δ*)/*μ*(*μ* + *ν* + *δ*), *Aν*/*μ*(*μ* + *ν* + *δ*)) is locally asymptotically stable. In addition, since model (4) is linear, by the theorems of stability of the differential equations, we obtain that the equilibrium (*A*(*μ* + *δ*)/*μ*(*μ* + *ν* + *δ*), *Aν*/*μ*(*μ* + *ν* + *δ*)) is globally asymptotically stable, which completes the proof.

Now, we compute the basic reproduction number of model (2) by applying the way given in [33, 34] by Wang et al. Let

(7) Ft,x=βtS+ϵVE+I000,Vt,x=λ+μEμ+fI−λEβtSE+I+μ+νS−A−δVμ+δV+ϵβtVE+I−νS,

where *x* = (*E*, *I*, *S*, *V*)^*T*^, and then model (2) takes the following form:

(8) $$\begin{array}{c} \dot{x}\left(\;t\;\right)=F\left(\;t,x\;\right)-V\left(\;t,x\;\right)≜f\left(\;t,x\left(\;t\;\right)\;\right). \end{array}$$

Obviously, model (8) has a disease-free equilibrium *x* ^*∗*^(*t*) = (0,0, *S* ^*∗*^, *V* ^*∗*^).

Next, we set two 2 × 2 matrices as follows:

(9) Ft=∂Fit,x∗t∂xj1≤i,j≤2,Vt=∂Vit,x∗t∂xj1≤i,j≤2,

where *ℱ*_*i*_(*t*, *x*(*t*)) and *𝒱*_*i*_(*t*, *x*(*t*)) are the *i*th component of *ℱ*(*t*, *x*(*t*)) and *𝒱*(*t*, *x*(*t*)), respectively. Then, by simple computations, it follows that

(10) Ft=βtS∗+ϵV∗βtS∗+ϵV∗00,Vt=λ+μ0−λμ+f.

Hence, we easily check that conditions (A1)–(A7) given in [33] are satisfied.

Let *Y*(*t*, *s*) be the 2 × 2 matrix solution of the following initial value problem:

(11) ddtYt,s=−VtYt,s∀t≥s,Ys,s=I.

Let *C*_*ω*_ be the ordered Banach space of all *ω*-periodic continuous function form *R* to *R* ^2^ with the maximum norm ‖·‖. The positive cone *C*_*ω*_^+^ = {*ϕ* ∈ *C*_*ω*_ : *ϕ*(*t*) ≥ 0  for  all  *t* ∈ *R*}. Suppose *ϕ*(*s*) ∈ *C*_*ω*_ is the initial distribution of infectious individuals in this periodic environment, then *F*(*s*)*ϕ*(*s*) is the rate of new infectious individuals produced by the infected individuals who were introduced at time *s*, and *Y*(*t*, *s*)*F*(*s*)*ϕ*(*s*) represents the distributions of those infected individuals who were newly infected at time *s* and remain in the infected compartment at time *t* for *t* ≥ *s*. Hence, we define a linear operator *L* : *C*_*ω*_ → *C*_*ω*_ as follows:

(12) Lϕt=∫0+∞Yt,t−aFt−aϕt−ada∀t∈R,  ϕ∈Cω.

The operator *L* is positive, continuous, and compact on *C*_*ω*_. Thus, *R*_0_ can be characterized by the existence of a nonnegative and nonzero *ϕ* ∈ *C*_*ω*_^+^ such that

(13) $$\begin{array}{c} \left(\;L\phi \;\right)\left(\;t\;\right)=R_{0}\phi \left(\;t\;\right). \end{array}$$

Now, we define basic reproduction number *R*_0_ for model (8) by

(14) $$\begin{array}{c} R_{0}=\rho \left(\;L\;\right), \end{array}$$

where *ρ*(*L*) is the spectral radius of *L*.

Using Theorem 2.2 given in [33], we can obtain the following results on basic reproduction number *R*_0_ and the locally asymptotical stability of disease-free equilibrium *P*_0_ for model (2).

**Lemma 2 .**

(*1*) On the basic reproduction number *R*_0_, one has

(i) *R*_0_ = 1 if and if only *ρ*(Φ_*F*−*V*_(*ω*)) = 1;
(ii) *R*_0_ > 1 if and if only *ρ*(Φ_*F*−*V*_(*ω*)) > 1;
(iii) *R*_0_ < 1 if and if only *ρ*(Φ_*F*−*V*_(*ω*)) < 1.

(*2*) *E* ^*∗*^(*t*) is locally asymptotically stable if *R*_0_ < 1 and unstable if *R*_0_ > 1.

Let, for any integer *n* > 0, *R*_+_^*n*^ = {(*x*_1_, *x*_2_,…, *x*_*n*_) ∈ *R* ^*n*^ : *x*_*i*_ ≥ 0, *i* = 1,2,…, *n*}. For *u*, *v* ∈ *R* ^*n*^, we denote *u* ≥ *v* if *u* − *v* ∈ *R*_+_^*n*^, *u* > *v* if *u* − *v* ∈ *R*_+_^*n*^∖{0}, and *u* ≫ *v* if *u* − *v* ∈ int⁡*R*_+_^*n*^, respectively, where int⁡*R*_+_^*n*^ denotes the interior of *R*_+_^*n*^.

Let *B*(*t*) be a continuous and *ω*-periodic *n* × *n* matrix function; we consider the following linear system:

(15) $$\begin{array}{c} \dot{x}=B\left(\;t\;\right)x. \end{array}$$

Let Φ_*B*_(*t*) be the fundamental solution matrix of system (15) with initial condition Φ_*B*_(0) = *I*, where *I* is *n* × *n* identity matrix and let *ρ*(Φ_*B*_(*ω*)) be the spectral radius of matrix Φ_*B*_(*ω*).

Further, we assume that *B*(*t*) also is cooperative and irreducible; then by the Perron-Frobenius theorem, *ρ*(Φ_*B*_(*ω*)) is the principle eigenvalue of Φ_*B*_(*ω*) in the sense that it is simple and admits an eigenvector *ν* ^*∗*^ ≫ 0.

**Lemma 3 (see [35]).**

Let *B*(*t*) be a continuous, cooperative, irreducible, and *ω*-periodic *n* × *n* matrix function; *μ* = (1/*ω*)ln⁡*ρ*(Φ_*B*_(*ω*)). Then there exists a positive *ω*-periodic function *ν*(*t*) such that *x*(*t*) = *e* ^*μt*^ *ν*(*t*) is a solution of system (15).

## 3. Main Result

**Theorem 4 .**

The disease-free equilibrium *P*_0_ of model (2) is globally asymptotically stable if *R*_0_ < 1 and unstable if *R*_0_ > 1.

**Proof**

From Lemma 2, we obtain that if *R*_0_ < 1, *P*_0_ is locally asymptotically stable and unstable if *R*_0_ > 1. Now, we will only prove the attractivity of *P*_0_ for the case *R*_0_ < 1. From *R*_0_ < 1 and conclusion (iii) of Lemma 2, we have *ρ*(Φ_*F*−*V*_(*ω*)) < 1, and then we can choose a small enough constant *η* > 0 such that *ρ*(Φ_*F*−*V*+*ηM*_(*ω*)) < 1, where

(16) $$\begin{array}{c} M\left(\;t\;\right)=\left(\begin{array}{cc} \beta \left(t\right)\left(1+\epsilon \right) & \beta \left(t\right)\left(1+\epsilon \right)\\ 0 & 0 \end{array}\right). \end{array}$$

By Lemma 1, we obtain that, for above given constant *η*, there exists *t*_1_ > 0 such that for all *t* > *t*_1_

(17) St≤S∗+η,Vt≤v∗+η.

From the second and third equations of model (1), we obtain that, for all *t* > *t*_1_,

(18) dEdt≤βtS∗+η+ϵV∗+ηE+I−λ+μE,dIdt=λE−μ+fI.

Consider the following auxiliary system:

(19) dE~dt=βtS∗+η+ϵV∗+ηE~+I~−λ+μE~,dI~dt=λE~−μ+fI~.

For convenience, we will rewrite it as follows:

(20) $$\begin{array}{c} \frac{d}{dt}\left(\begin{array}{c} \tilde{E}\\ \tilde{I} \end{array}\right)=\left(\;F\left(\;t\;\right)-V\left(\;t\;\right)+\eta M\left(\;t\;\right)\;\right)\left(\begin{array}{c} \tilde{E}\\ \tilde{I} \end{array}\right). \end{array}$$

From Lemma 3, it follows that there exists a positive *ω*-periodic function *q*(*t*) = (*q*_1_(*t*), *q*_2_(*t*))^*T*^ such that $\left(\tilde{E}(t),\tilde{I}(t){)}^{T}=e^{\xi t}q(t\right)$ is a solution of model (19), where *ξ* = (1/*ω*)ln⁡(*ρ*(Φ_*F*−*V*+*ηM*_(*ω*))).

Denote *J*(*t*) = (*E*(*t*), *I*(*t*))^*T*^. We can choose a small constant *θ* > 0 such that *J*(*t*_1_) ≤ *θq*(*t*_1_). Then, from (18) the comparison principle implies that

(21) $$\begin{array}{c} J\left(\;t\;\right)\leq \theta e^{\xi t}q\left(\;t\;\right)\;\forall t>t_{1}. \end{array}$$

By *ρ*(Φ_*F*−*V*+*ηM*_(*ω*)) < 1, it follows that *ξ* < 0, then lim_*t*→*∞*_⁡*J*(*t*) = 0, and, that is,

(22) limt→∞⁡Et=0,limt→∞⁡It=0.

Moreover, from the equations of model (2), we can get

(23) limt→∞⁡St=S∗,limt→∞⁡Vt=V∗.

Hence, disease-free equilibrium *P*_0_ of model (2) is globally attractive. This completes the proof.

## 4. Model Applications

### 4.1. Parameters Estimation and Simulation Results

**Figure 5:**
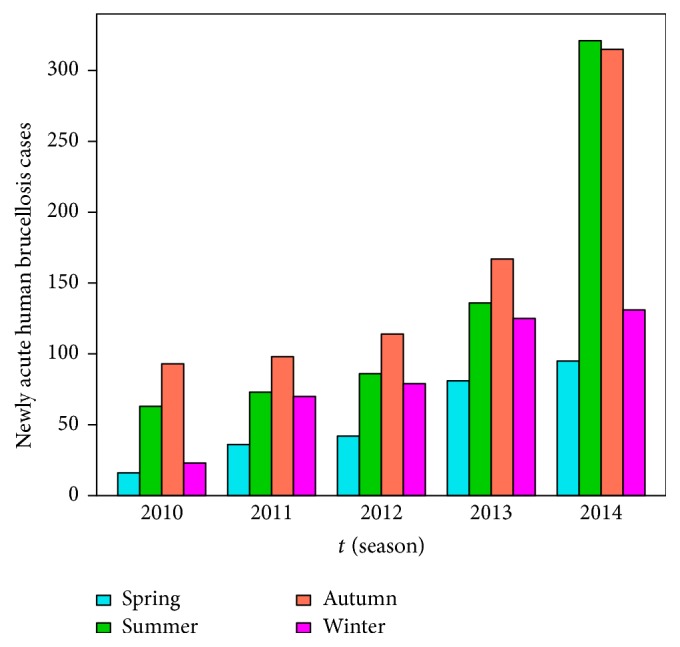
The reported number of newly acute human brucellosis cases from 2010 to 2014.

In this section, we apply model (1) to simulate the reported data of newly acute human brucellosis cases in Bayingolin Mongol Autonomous Prefecture in each season. We only use the reported data of newly acute human brucellosis cases from 2010 to 2014 (see Figure 5) because of the fact that the reported data from 2005 to 2009 is relatively flat and has not shown a gradual increasing trend. However, it has an influence on the simulation for the rapid growth number of newly acute human brucellosis cases from 2010 to 2014. In the summer and autumn of 2014, the human brucellosis experienced an outbreak; thus the reported number of newly acute human brucellosis cases increased dramatically in that year.

The parameter values of model (1) are listed in Table 2, and we interpret the parameter values as follows.

[A] From* Bayingolin Mongol Autonomous Prefecture Statistical Yearbook 2014*, we obtain the annual birth populations and natural mortality rate and divide them by 4 to derive the birth populations *A*, *B* and natural mortality rates *μ*, *μ*_1_.
[B] The mean incubation period of brucellosis is almost two weeks [36] and about 1/6 quarter, so we have *λ* = 6.
[C] The acute infection period of human brucellosis is about a half of a year [36] and approximately 2 quarters, so we have *ω*_*h*_ = 1/2 = 0.5.
[D] In Xinjiang, vaccine* B. suis* strain 2 has been used to control brucellosis. The immune duration of vaccine* B. suis* strain 2 is about 2.5 years [23], similar to 10 quarters, so we have *δ* = 1/10 = 0.1.
[E] We did not acquire the deterministic information of sheep/cattle invalid vaccination rate *ϵ* in Bayingolin Mongol Autonomous Prefecture. However, vaccine* B. suis* strain 2 can protect 82% of sheep/cattle from* Brucella* annually in Inner Mongolia [37], and we use it as a substitute for *ϵ* = (1 − 0.82)/4.

We assume that the slaughter rate of infected sheep/cattle *f* and the vaccination rate of susceptible sheep/cattle *ν* in Bayingolin Mongol Autonomous Prefecture are approximate to the whole Xinjiang region, respectively. Thus we can suppose that *f* = 0.0983 and *ν* = 0.0412 [38]. In humans, the cure rate from the acute infection to the susceptible *η* = 0.6 and the transfer rate from the acute infection to the chronic infection 1 − *η* = 0.4 are given by [31].

We need the initial values to perform the numerical simulations of model (1). We determine both initial values *S*(0) = 944500 and *S*_*h*_(0) = 292710 from the* Bayingolin Mongol Autonomous Prefecture Statistical Yearbook*. The initial value *I*_ha_(0) = 16 is obtained from the Center for Disease Control and Prevention of Bayingolin Mongol Autonomous Prefecture in Xinjiang. We can derive the initial value *V*(0) from *S*(0) by vaccination rate *ν* and deduce the initial value *E*(0) from the initial values *S*(0) and *V*(0) by the parameters *ϵ*, *β*(*t*). Similarly, we can derive the initial value *I*(0) from *E*(0) by the parameter *λ* and deduce the initial value *I*_hc_(0) from *I*_ha_(0) by the parameter *ω*_*h*_(1 − *η*). Above initial values are *E*(0) = 3000, *I*(0) = 2500, *V*(0) = 377800, *I*_hc_(0) = 7.

**Figure 6:**
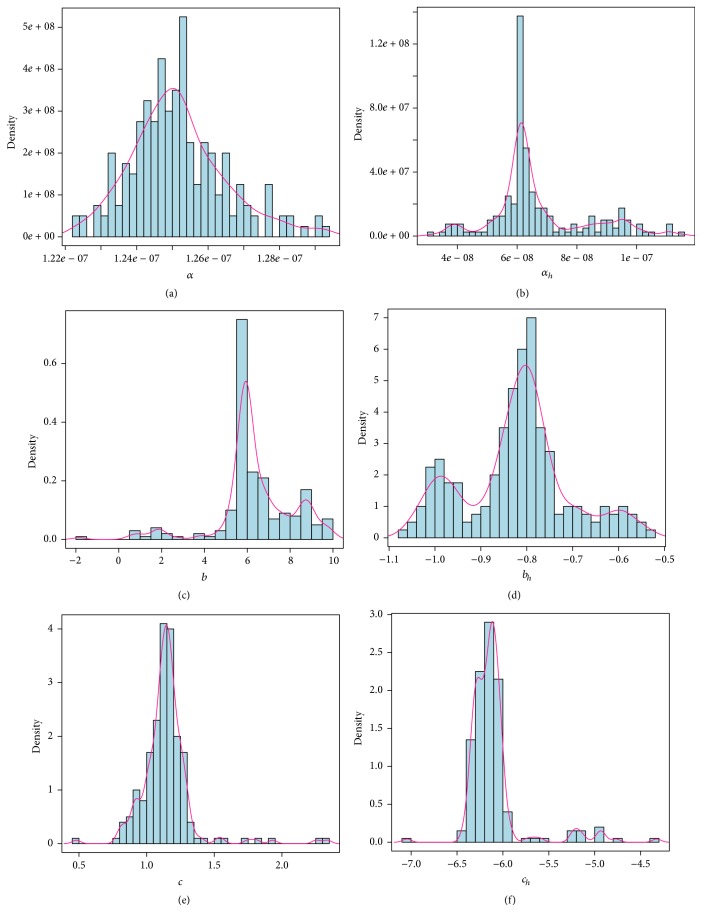
Frequency distribution histograms and probability density curves of the estimated parameters *α*, *b*, *c*, *α*_*h*_, *b*_*h*_, *c*_*h*_. The blue bars represent frequency distribution histograms and pink lines represent probability density curves.

For the functions of both periodic transmission rate functions *β*(*t*) = *α*[1 + *b*sin⁡((*π*/2)*t* + *c*)] and *β*_*h*_(*t*) = *α*_*h*_[1 + *b*_*h*_sin⁡((*π*/2)*t* + *c*_*h*_)], we use the least-square fitting and bootstrap sampling method to estimate the six parameters *α*, *b*, *c*, *α*_*h*_, *b*_*h*_, and *c*_*h*_ and the 95% confidence interval for each parameter which are listed in Table 3. Hence, two periodic transmission rates functions are expressed as *β*(*t*) = 1.2507 × 10^−7^[1 + 5.9830sin⁡((*π*/2)*t* + 1.14029)] and *β*_*h*_(*t*) = 6.1790 × 10^−8^[1 − 0.8115sin⁡((*π*/2)*t* − 6.1382)]. According to the bootstrap estimate value for each parameter, we plot the frequency distribution histogram and the probability density curve which are presented in Figure 6.

**Figure 7:**
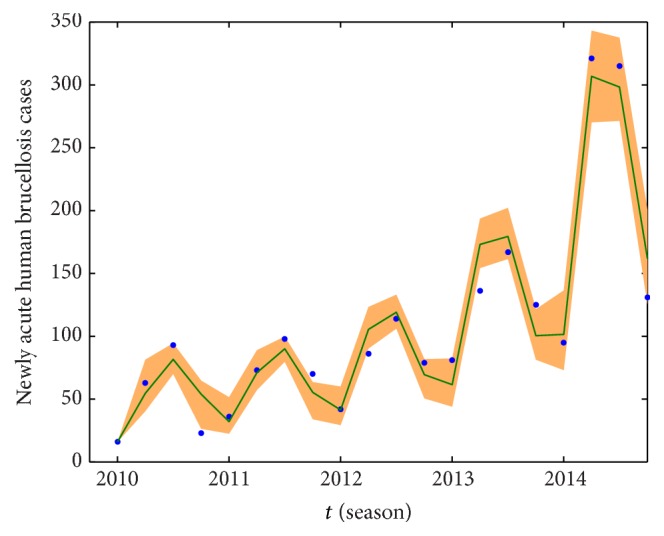
The number of newly acute human brucellosis cases and fitted curve. The blue dots represent the real data while the dark green solid curve is fitted by using our model, and the salmon area represents the 95% confidence interval around model fitted.

**Figure 8:**
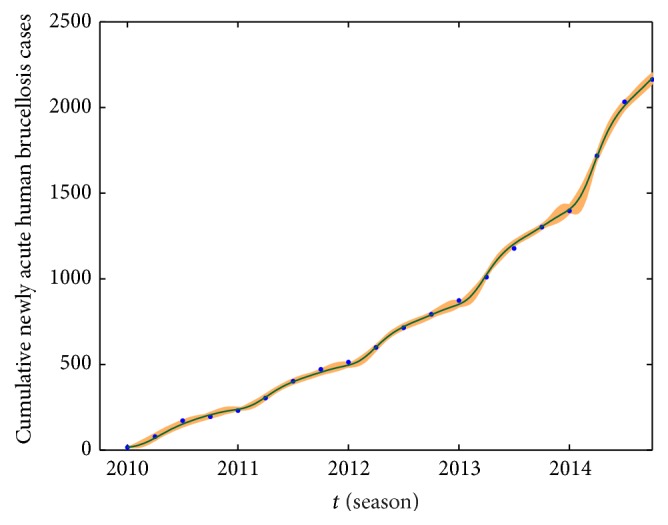
The cumulative number of newly acute human cases and fitted curve. The blue dots represent the real data while the dark green solid curve is fitted by using our model, and the salmon area represents the 95% confidence interval around model fitted.

We take the spring of 2010 as the start time of simulation, and the numerical simulations of the model on the number of newly acute human brucellosis cases in each season are presented in Figure 7. Moreover, under the same conditions which include parameter values and initial values, the cumulative numbers of newly acute human brucellosis cases and fitted curve are presented in Figure 8. At the same time, we estimated the 95% confidence interval for fitted curves by the bootstrap sampling method with salmon areas which are presented in Figures 7 and 8, respectively. It indicates that our model provides good matches to the reported data from Figures 7 and 8.

The mean absolute percentage error (MAPE) and the root mean square percentage error (RMSPE) are critical evaluation indicators, which are used to assess the fitting effect and the precision of our established model. The MAPE and the RMSPE are defined as

(24) MAPE=1n∑q=2nWq∗−WqWq∗×100%,RMSPE=∑q=2nWq∗−Wq/Wq∗2n−1×100%,

where *W*(*q*)^*∗*^ is the real value at time *q* and *W*(*q*) is its fitting value and *n* is the number of data used for prediction. The criteria of MAPE and RMSPE are shown in Table 4 [39, 40]. We use model (1) to simulate the number of newly acute human brucellosis cases in each season, where MAPE = 18.07% and RMSPE = 20.89%. When we simulate the cumulative number of newly acute human brucellosis cases, the values of MAPE and RMSPE are 2.55% and 4.03%, respectively. Comparing with the criteria of MAPE and RMSPE, the real data and the fitted curve match quite well by using our model.

### 4.2. Model Predication

**Figure 9:**
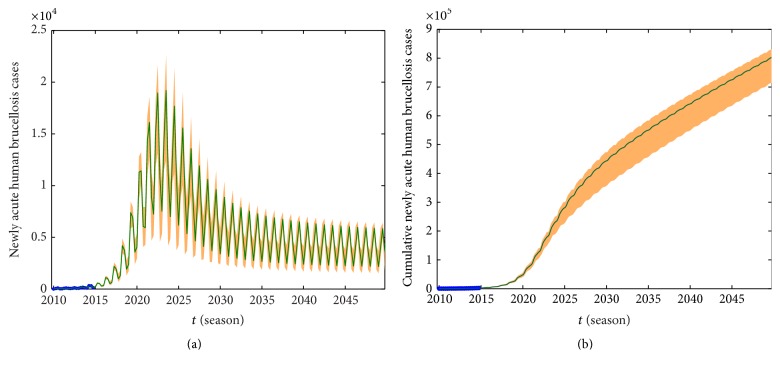
Prediction of newly acute human brucellosis in 35 years from 2015 to 2049. (a) The number of newly acute human brucellosis cases. (b) The cumulative number of newly acute human brucellosis cases.

We can not only fit the real data by using our model but also predict the fluctuation tendency in the next 35 years about 140 quarters which are presented in Figure 9(a). The predicted values and 95% confidence interval for each season from 2015 to 2034 are listed in Table 5. Combining Figure 9(a) with Table 5, we find that human brucellosis increases sharply from the spring of 2015 to the winter of 2023 and reaches the peak 15325 [95% CI: 11920–18242] in the summer of 2023. Shortly afterwards, the number of newly acute human brucellosis starts to gradually reduce during the period of 2023 to 2040 and maintains its equilibrium level after 2040.  Figure 9(b) describes the predicted tendency of cumulative number of newly acute human brucellosis from 2015 to 2049.

### 4.3. The Calculation of Basic Reproduction Number

According to the method for basic reproduction number with periodic coefficients (see [33, Theorem 2.1]), we can calculate the basic reproduction number *R*_0_ = 2.5524 [95% CI: 2.5129–2.6225] which means that human brucellosis in Bayingolin Mongol Autonomous Prefecture persists under current circumstances. When model (2) degenerates into an autonomous case with $\bar{\beta }=(1/4)\int_{0}^{4}\beta (t)dt$, we obtain

(25) F=β¯S∗+ϵV∗β¯S∗+ϵV∗00,V=λ+μ0−λμ+f.

**Figure 10:**
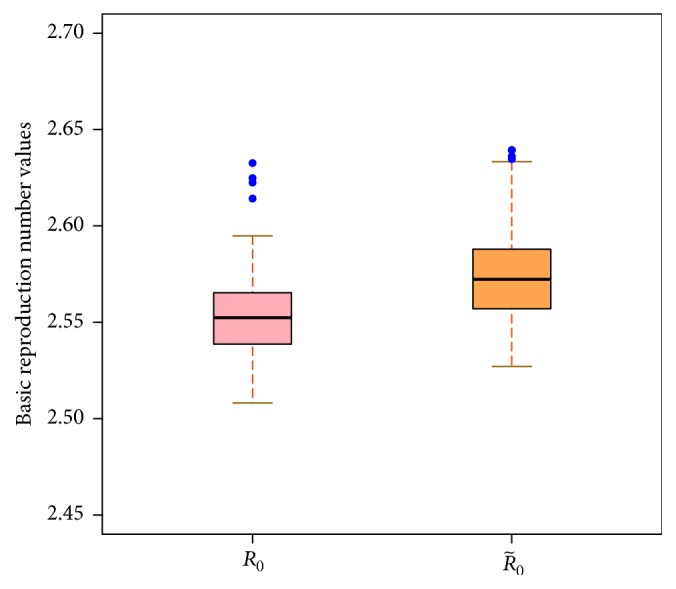
The boxplots of estimated basic reproduction number *R*_0_ and average basic reproduction number ${\tilde{R}}_{0}$.

**Figure 11:**
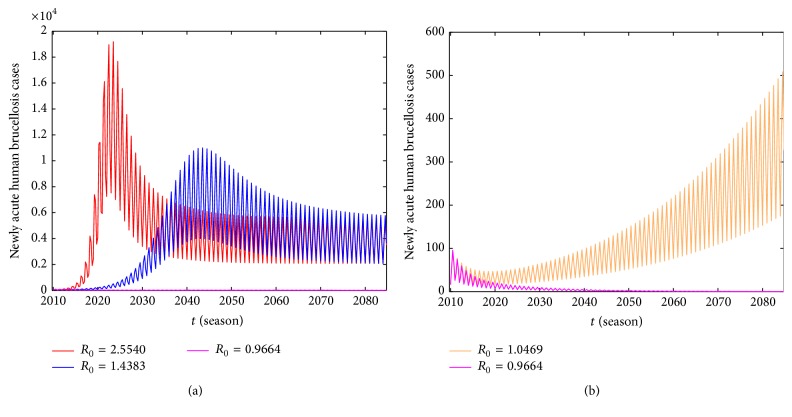
The variations of the newly acute human brucellosis cases for different values of *R*_0_. (a) When *ν* = 0.0412, 0.206, and 0.412, *R*_0_ = 2.554, 1.4383, and 0.9664, respectively. (b) When *ν* = 0.36, *R*_0_ = 1.0469. Other parameters are as in Tables 2 and 3.

Using the method given by van den Driessche and Watmough given in [34], we obtain basic reproduction number

(26) $$\begin{array}{c} {\tilde{R}}_{0}=\frac{\bar{\beta }A\left(f+\lambda +\mu \right)\left(\delta +\mu +\epsilon \nu \right)}{\mu \left(f+\mu \right)\left(\lambda +\mu \right)\left(\delta +\mu +\nu \right)}, \end{array}$$

which is called the average basic reproduction number. Meanwhile we also estimate ${\tilde{R}}_{0}=2.5723$ [95% CI: 2.5671–2.6393]. The boxplots for *R*_0_ and ${\tilde{R}}_{0}$ are presented in Figure 10. We can see that ${\tilde{R}}_{0}$ is slightly higher than *R*_0_, and it implies utilizing average method to calculate ${\tilde{R}}_{0}$ which overestimates the risk of epidemic of human brucellosis. Moreover, we demonstrate that the periodic basic reproduction number *R*_0_ is a threshold, which determines whether or not brucellosis persists in the population. From Figures 11(a) and 11(b), it is clear that when *R*_0_ < 1, the number of newly acute human brucellosis tends to zero. On the contrary, when *R*_0_ > 1, the number of newly acute human brucellosis tends to be a stable periodic solution. Human brucellosis cases increase with the raise of the basic reproduction number *R*_0_.

### 4.4. Sensitivity Analysis and Disease Control

**Figure 12:**
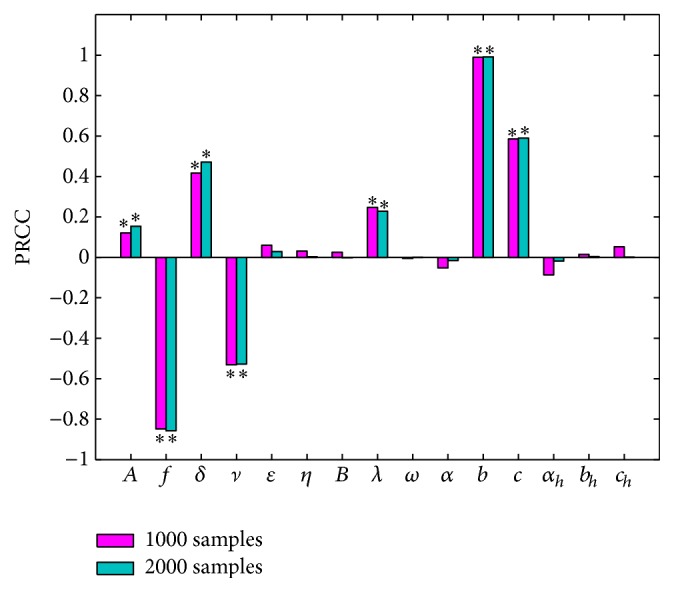
Partial rank correlation coefficients (PRCC) results for the dependence of newly acute human brucellosis cases in each season on each parameter. *∗* denotes the value of PRCC which is not zero significantly, where the significance level is 0.05.

We use Latin hypercube sampling (LHS) and partial rank correlation coefficients (PRCC) [41] to examine parameters which have a significant influence on the number of newly acute human brucellosis cases. We choose the sample size *n* = 1000 and *n* = 2000, respectively, parameters interested as the input variables, and the number of newly acute human brucellosis cases as the output variable. The accurate PRCC values and *p* values of each parameter with sample size *n* = 1000 and *n* = 2000 are listed in Table 6, respectively.  Figure 12 depicts the PRCC values of each parameter, we assume the significance level *α* = 0.05, and parameters with star above the bar are the significant ones. Combining Table 6 with Figure 12, we find that there is no significant difference between the PRCC values and *p* values when comparing the sampling size *n* = 1000 with *n* = 2000. The larger the PRCC in absolute value, the more important the parameters in responding to the change of newly acute human brucellosis cases; therefore we can confirm that parameters *A*, *δ*, *λ*, *b*, *c* have positive impact on the number of newly acute human brucellosis cases. On the contrary, *f* and *ν* have negative impact. We do not take the other parameters into account due to the reason that PRCC values are small and *p* > 0.05.

**Figure 13:**
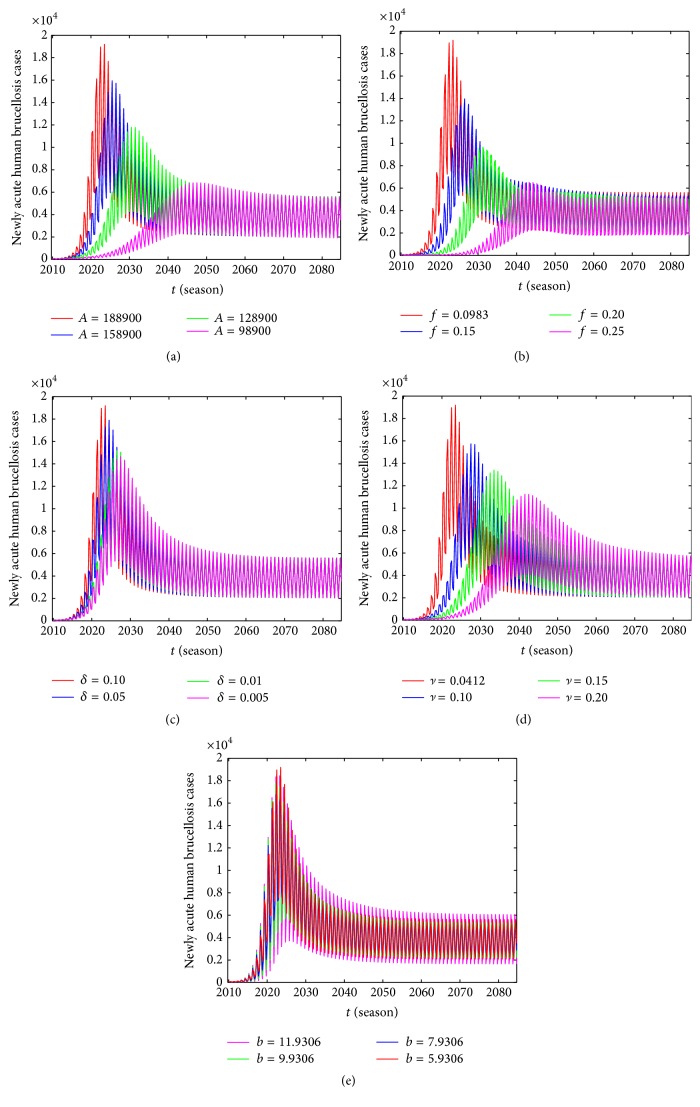
The influence of different parameters on the number of newly acute human brucellosis cases *I*_ha_(*t*): (a) different values of *A*; (b) different values of *f*; (c) different values of *δ*; (d) different values of *ν*; (e) different values of *b*.

Through the above mentioned analysis, we demonstrate that parameters *A*, *δ*, *λ*, *b*, *c*, *f*, *ν* have significant impact on the number of newly acute human brucellosis cases, so it is necessary to study the influence of parametric modification on the human brucellosis epidemic which are presented in Figure 13. We can see that the effects of parameters *A*, *f*, *ν* are stronger and other parameters have little impact on the newly acute human brucellosis cases; moreover, we find that the parameter changes can influence not only the number of newly acute human brucellosis cases but also the time of peak for newly acute human brucellosis cases. As Figures 13(a) and 13(c) illustrated when fixing other parameters at constant, the number of newly acute human brucellosis cases falls substantially with a decrease in *A* and *δ*, respectively. And the peak of initial outbreak will be postponed. As similar as above, we investigate the impact of parameters *f* and *ν* on newly acute human brucellosis cases which are presented in Figures 13(b) and 13(d), respectively. We observe that newly acute human brucellosis cases decrease with an increase in *f* and *ν*, and the peak of initial outbreak will be postponed. From Figure 13(e) we can see that parameter *b* does not have such effects; it only controls the magnitudes of forcing. We do not consider the influence of parameters *λ* and *c* on newly acute human brucellosis cases for the reason that *λ* is expressed as the transfer rate from exposed to infected class in sheep/cattle, and *c* denotes phase.

Finally, in order to find better control strategies for brucellosis transmission, we carry out some sensitivity analysis to confirm the influence of parameters *A*, *f*, *δ*, *ν* on *R*_0_.

**Figure 14:**
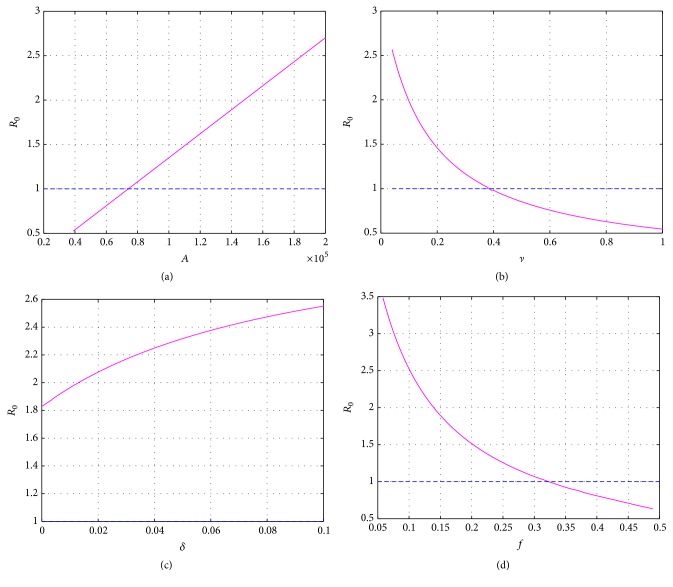
The influence of parameters on *R*_0_: (a) versus A; (b) versus *ν*; (c) versus *δ*; (d) versus *f*.

We show variations of *R*_0_ for different values of *A* in Figure 14(a), which illustrates that parameter *A* has a great impact on *R*_0_, the values of *R*_0_ increase as *A* is rising, and there appears a linear relationship. When *A* is less than 74100, *R*_0_ < 1, the disease can die out. Nevertheless, at present, the birth number of sheep/cattle can achieve 188900 in Bayingolin Mongol Autonomous Prefecture in each season. This indicates that, in order to eradicate human brucellosis, herdsmen should reduce the birth number of sheep/cattle without affecting the economic benefit.

It is well known that vaccination for the susceptible sheep/cattle is an effective measure to control brucellosis. The influence of *ν* on *R*_0_ is given in Figure 14(b). It can be observed that the value of *R*_0_ decreases as *ν* is increasing. Moreover, Figure 14(b) shows that when vaccination rate *ν* is higher than 0.375, *R*_0_ < 1, the disease can be eradicated. Thus, government should strengthen the vaccination rate of susceptible sheep/cattle and improve the herd immunity level.  Figure 14(c) reflects that reducing the sheep/cattle loss of vaccination rate is also an approach to decrease *R*_0_. However, *R*_0_ cannot become less than one even if the sheep/cattle loss of immunity rate *δ* is zero. In other words, implementing this measure alone cannot eliminate brucellosis.

In fact, the common ways to dispose the infected sheep/cattle are slaughter and then bury.  Figure 14(d) represents the relationship between the slaughter rate *f* and *R*_0_. The value of *R*_0_ decreases as *f* is increasing. When *f* is higher than 0.32, *R*_0_ < 1, the disease can be eliminated. In real life, a large number of infected sheep/cattle are not culled, and the slaughter rate only reaches 0.0983 since enhancing the slaughter rate of infected sheep/cattle can inflict the most economic damage on the herdsman.

The above analysis demonstrates that human brucellosis can be controlled with three strategies: reducing the seasonal crop of newborn sheep/cattle, increasing the vaccination rate of susceptible sheep/cattle, and raising the slaughter rate of infected sheep/cattle.

## 5. Conclusion and Discussion

As a zoonotic disease, brucellosis is one of the biggest public health threats which cannot be ignored in China. Despite its acknowledgment as an important economic and health problem and the availability of proven control measures, it continues to occur with a relatively high frequency.

In this article, in order to explore effective control and prevention measures, by using the seasonal newly acute human brucellosis cases from 2010 to 2014 in Bayingolin Mongol Autonomous Prefecture of Xinjiang, we proposed an SEIV model with periodic transmission rates to investigate the spread of brucellosis. The model describes the transmission of brucellosis among sheep/cattle and from sheep/cattle to humans.

We estimated the basic reproduction number *R*_0_ = 2.5524 [95% CI: 2.5129–2.6225] and the average basic reproduction number ${\tilde{R}}_{0}=2.5723$ [95% CI: 2.5671–2.6393]. It shows that the average basic reproduction number ${\tilde{R}}_{0}$ overestimates the risk of brucellosis infection, and brucellosis persists with the current prevention and control measures in Bayingolin Mongol Autonomous Prefecture of Xinjiang. Hou et al. [23] estimated the basic reproduction number *R*_0_ = 1.8 in Inner Mongolia from 2005 to 2010. Li et al. [24, 31] calculated the basic reproduction number *R*_0_ = 1.9789 in Hinggan League of Inner Mongolia from 2001 to 2011. Nie et al. [22] obtained the basic reproduction number *R*_0_ = 1.1987 from 1987 to 1998 and *R*_0_ = 2.1327 from 1998 to 2005 in Jilin Province. That is to say, the dynamics of brucellosis in Inner Mongolia, Xinjiang, and Jilin of China are serious in these years. We need to control the disease spreading by carrying out effective measures.

Then we used our model to simulate the number of seasonal newly acute human brucellosis cases and predicted the general tendency of disease in Bayingolin Mongol Autonomous Prefecture. Next, we carried out some sensitivity analysis of newly acute human brucellosis cases and the basic reproduction number *R*_0_ in terms of the model parameters which have significant influence on the number of newly acute human brucellosis cases by partial rank correlation coefficients (PRCC).

Finally, there are some limitations in this research. Firstly, the influence of* Brucella* in the environment was not taken into account, and it maybe affects the whole dynamic model for brucellosis transmission. Secondly, other common animals also can transmit the brucellosis to humans such as dogs, pigs, and horse. We leave these for further research.

## Figures and Tables

**Table 1:** The season exponent of average newly acute human brucellosis cases from 2005 to 2014.

Season	Average newly acute human brucellosis cases (${\bar{x}}_{j}$)	Season exponent (*S*_*j*_)
Spring	38.6	0.56
Summer	91.2	1.32
Autumn	96.1	1.39
Winter	50.8	0.73

	$\bar{x}$ = 69.71	

**Table 2:** The parameter values and interpretations.

Parameter	Unit	Value	Comments	Reference
*A*	season^−1^	188900	Sheep/cattle birth population	[A]
*μ*	season^−1^	0.05	Sheep/cattle natural mortality rate	[A]
*f*	season^−1^	0.0983	The slaughter rate of infected sheep/cattle	Assumption
*δ*	season^−1^	0.1	Sheep/cattle loss of vaccination rate	[D]
*ν*	season^−1^	0.0412	The vaccination rate of susceptibility sheep/cattle	Assumption
*ϵ*	season^−1^	0.0450	Sheep/cattle invalid vaccination rate	[E]
*λ*	season^−1^	6	Transfer rate from exposed to infected	[B]
*B*	season^−1^	3305	Human birth population	[A]
*η*	season^−1^	0.6	The cure rate from acute infection to susceptible	[31]
1 − *η*	season^−1^	0.4	Transfer rate from acute infection to susceptible	[31]
*ω*_*h*_	season^−1^	0.5	The leave rate from acute infection	[C]
*μ*_1_	season^−1^	0.0010	Human natural mortality rate	[A]

**Table 3:** Parameter values for point estimation and 95% interval estimation.

Parameter	Point estimation	95% confidence interval
*α*	1.2507 × 10^−7^	[1.2290 × 10^−7^, 1.2821 × 10^−7^]
*b*	5.9830	[1.7072, 9.5677]
*c*	1.14029	[0.83036, 1.5590]
*α*_*h*_	6.1790 × 10^−8^	[3.8848 × 10^−8^, 1.0290 × 10^−7^]
*b*_*h*_	−0.8115	[−1.0351, −0.5770]
*c*_*h*_	−6.1382	[−6.3661, −4.9694]

**Table 4:** Criteria of MAPE and RMSPE.

MAPE and RMSPE	Forecasting power
<10%	Highly accurate forecasting
10–20%	Good forecasting
20–50%	Reasonable forecasting
>50%	Inaccurate forecasting

**Table 5:** The predicted number of newly acute human brucellosis cases from the spring of 2015 to the winter of 2034 and 95% confidence interval.

Year	Spring	Summer	Autumn	Winter
2015	181 [131–246]	575 [487–652]	533 [478–604]	282 [211–373]
2016	340 [247–469]	1118 [928–1287]	1004 [887–1163]	521 [377–735]
2017	659 [482–919]	2195 [1818–2543]	1943 [1697–2276]	998 [700–1458]
2018	1281 [938–1784]	4193 [3464–4863]	3750 [3258–4292]	1921 [1319–2775]
2019	2393 [1749–3239]	7388 [5866–8439]	6903 [6023–7943]	3572 [2404–4762]
2020	4064 [2957–5341]	11290 [8706–12826]	11431 [10142–13223]	6057 [4085–7929]
2021	5935 [4283–7929]	14361 [10993–16722]	16113 [12757–18557]	8839 [4684–11153]
2022	7244 [5179–9825]	14415 [11556–17446]	18959 [13194–21629]	10786 [4582–13018]
2023	7526 [5339–10324]	15325 [11920–18242]	19191 [12443–22493]	11260 [4198–13130]
2024	7008 [4864–10170]	12669 [10336–15563]	17674 [11259–21278]	10606 [3758–12407]
2025	6175 [4253–9571]	10878 [8868–13556]	15574 [10089–18989]	9489 [3367–11094]
2026	5350 [3707–8676]	9365 [7607–11835]	13572 [9104–16576]	8349 [3053–9760]
2027	4657 [3270–7775]	8186 [6609–10483]	11908 [8332–14458]	7365 [2813–8590]
2028	4114 [2891–6989]	7297 [5857–9463]	10605 [7749–12739]	6578 [2635–7625]
2029	3702 [2603–6349]	6634 [5291–8708]	9612 [7319–11391]	5968 [2504–6861]
2030	3393 [2388–5842]	6141 [4856–8151]	8862 [7003–10349]	5504 [2408–6267]
2031	3160 [2408–6267]	5771 [4524–7739]	8297 [6770–9546]	5152 [2337–5821]
2032	2985 [2104–5142]	5490 [4276–7430]	7867 [6596–8926]	4883 [2283–5484]
2033	2851 [2010–4903]	5271 [4065–7192]	7536 [6431–8443]	4676 [2239–5224]
2034	2746 [1937–4715]	5097 [3904–7028]	7276 [6272–8087]	4514 [2202–5019]

**Table 6:** Partial rank correlation coefficients (PRCC) for newly acute human brucellosis cases in each season and each input parameter variable.

Input parameter	1000 samples		2000 samples	
	PRCC	*p* value	PRCC	*p* value
*A*	0.1208	1.426 × 10^−4^	0.1541	5.0505 × 10^−12^
*f*	−0.8482	7.538 × 10^−274^	−0.8577	0
*δ*	0.4172	8.2104 × 10^−43^	0.4712	2.623 × 10^−110^
*ν*	−0.5313	6.4607 × 10^−73^	−0.5273	1.4028 × 10^−142^
*ϵ*	0.0601	0.0590	0.0288	0.19967
*η*	0.0318	0.3184	0.003	0.8854
*B*	0.0249	0.4347	−0.002	0.9146
*λ*	0.2469	3.6347 × 10^−15^	0.2279	0.8100 × 10^−25^
*ω*	−0.004	0.8790	0.001	0.9620
*α*	−0.0522	0.1012	−0.0159	0.4764
*b*	0.9895	0	0.9913	0
*c*	0.5856	7.734 × 10^−92^	0.5897	2.5675 × 10^−186^
*α*_*h*_	−0.0866	0.0647	−0.0183	0.4151
*b*_*h*_	0.0151	0.6349	0.003	0.8779
*c*_*h*_	0.0532	0.0961	0.001	0.9560
